# Repeated tracheal tube damage due to an ultrasonic bone aspirator in Le Fort I osteotomy

**DOI:** 10.1186/s40981-022-00570-z

**Published:** 2022-10-04

**Authors:** Taiga Kobayashi, Mariko Muto, Chiaki Nemoto, Manabu Endo, Midori Mogami, Youichi Tanaka, Satoki Inoue

**Affiliations:** 1 The Junior Resident Center, Ohara General Hospital, 6-1 Ohomachi, Fukushima, 960-8611 Japan; 2 Department of Anesthesiology, Ohara General Hospital, 6-1 Ohomachi, Fukushima, 960-8611 Japan; 3 Department of Dentistry and Oral Surgery, Ohara General Hospital, 6-1 Ohomachi, Fukushima, 960-8611 Japan; 4 Department of Anesthesiology, Fukushima Medical University, 1 Hikarigaoka, Fukushima, Fukushima 960-1295 Japan

**Keywords:** Ultrasonic bone aspirator, Tracheal tube damage, Le fort I osteotomy

To the Editor:

Nasotracheal intubation is generally chosen for airway management during orofacial surgery [1]. We experienced an unusual case of repeated tracheal tube damage during Le Fort I osteotomy.

A 26-year-old woman (height, 155 cm; weight, 54 kg) presented with jaw deformities and was scheduled for Le Fort I osteotomy under general anesthesia. A reinforced tube was inserted from the right nostril with no difficulties and fixed at a depth of 26 cm. Using an ultrasonic bone aspirator (Sonopet®; Stryker, Kalamazoo, MI, USA), the surgeon split the maxillary bone near the lateral nasal wall. The surgeon thereafter noted air leakage around the endotracheal tube cuff. We attempted to inflate the cuff of the tracheal tube, but the pilot balloon did not work. Thus, we exchanged the tracheal tube and found that it was damaged 23 cm from the tip (Fig. 1). About 20 min after the operation was restarted, air leakage around the endotracheal tube cuff recurred. Thus, we were again required to exchange the tracheal tube. Both tracheal tube exchanges were smoothly performed using a video laryngoscope. We found that the tube was slightly melted at the same site at which the first tracheal tube had been damaged. By close attention to the surgical field after the maxillary bone was completely split, we observed the tracheal tube wall through a 1-cm laceration at the lateral nasal wall that had been inadvertently created while splitting the maxillary bone (Fig. 2). The surgery was completed with no further complications. The patient was transferred to the high care unit and then discharged from the hospital on postoperative day 10.

**Fig. 1:**
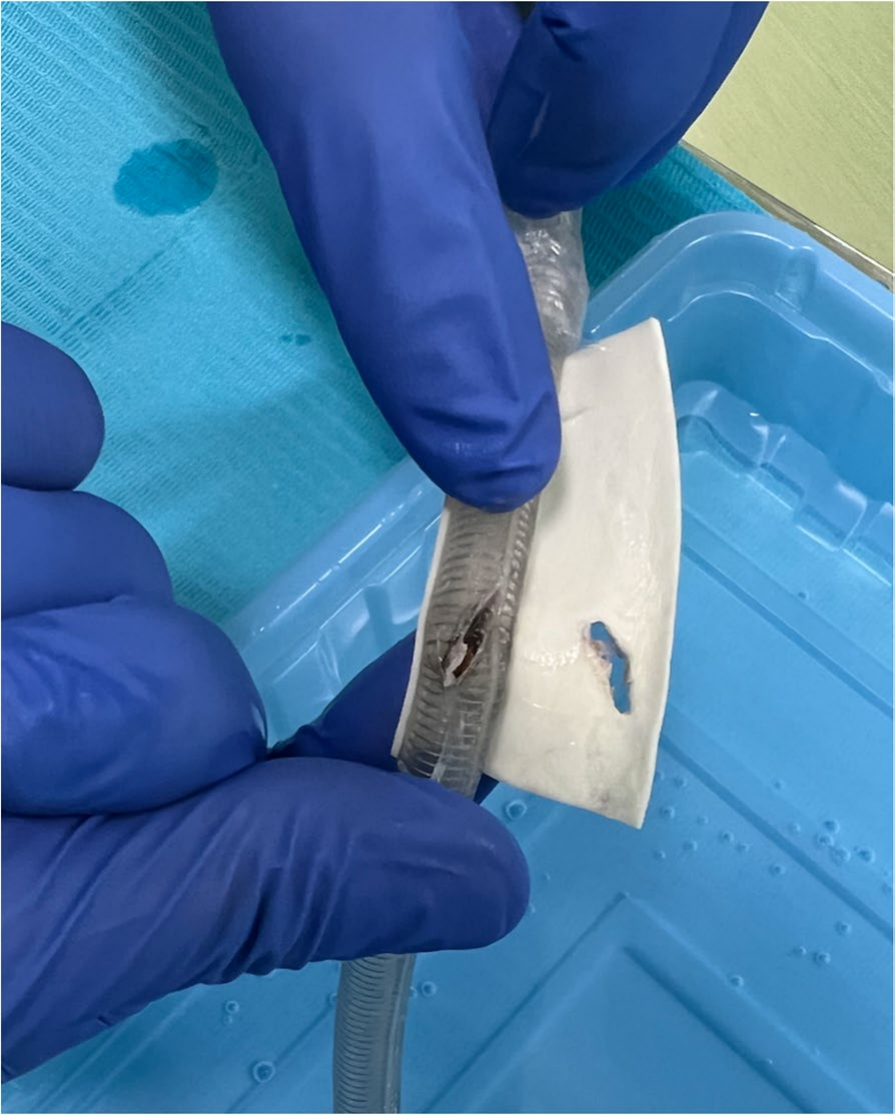
Injured tracheal tube. An approximately 1-cm burnt fissure was found 23 cm from the tip of the endotracheal tube. The fissure was located immediately behind the lateral nasal wall. To prevent a pressure ulcer in the nostril, the endotracheal tube was coated with a soft tape, which was also slashed by an ultrasonic bone aspirator

**Fig. 2:**
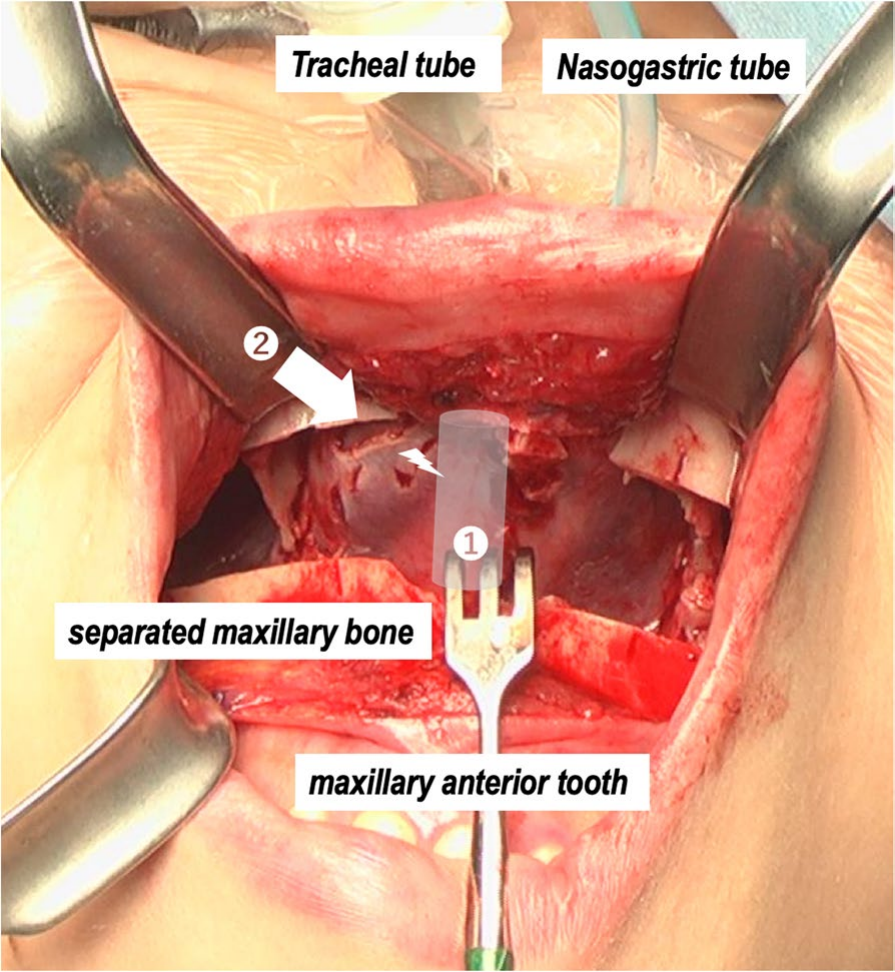
Image of the oral cavity after maxillary osteotomy with nasotracheal intubation. The nasal cavity was separated from the maxillary bone. Passage of the endotracheal tube was just behind the nasal bottom ①. At the lateral nasal wall, an approximately 1-cm laceration opened to the nasal cavity ②. Through this laceration, an ultrasonic bone aspirator inadvertently damaged the endotracheal tube

A nasotracheal tube does not present in the surgical field when splitting the maxillary bone through the Le Fort I osteotomy line. In this case, however, the lateral nasal wall was inadvertently injured, and a small laceration was made while splitting the maxillary bone. We considered that repeated tracheal tube damage occurred through this small laceration of the lateral nasal wall. It was probably very difficult for the surgeon to recognize that direct damage was occurring to the tracheal tube because the tracheal tube was not expected to be present at that location. This may explain why repeated tracheal tube damage occurred. There have been several reports regarding accidental nasotracheal tube injury during maxillofacial surgeries [2]. It might have been more difficult for our surgeon to notice the tube injury during the use of ultrasonic bone aspirator compared with conventional reciprocating saw. Therefore, it is important to be aware of the risk of nasotracheal tube damage during surgical procedures around the nasal wall bone. Conversely, the occurrence of nasotracheal tube damage during orofacial surgery can be considered a sign of unintentional nasal wall bone damage.

## Data Availability

Not applicable.
